# Ethanol confers differential protection against generalist and specialist parasitoids of *Drosophila melanogaster*

**DOI:** 10.1371/journal.pone.0180182

**Published:** 2017-07-12

**Authors:** Zachary R. Lynch, Todd A. Schlenke, Levi T. Morran, Jacobus C. de Roode

**Affiliations:** Department of Biology, Emory University, Atlanta, Georgia, United States of America; Biomedical Sciences Research Center Alexander Fleming, GREECE

## Abstract

As parasites coevolve with their hosts, they can evolve counter-defenses that render host immune responses ineffective. These counter-defenses are more likely to evolve in specialist parasites than generalist parasites; the latter face variable selection pressures between the different hosts they infect. Natural populations of the fruit fly *Drosophila melanogaster* are commonly threatened by endoparasitoid wasps in the genus *Leptopilina*, including the specialist *L*. *boulardi* and the generalist *L*. *heterotoma*, and both wasp species can incapacitate the cellular immune response of *D*. *melanogaster* larvae. Given that ethanol tolerance is high in *D*. *melanogaster* and stronger in the specialist wasp than the generalist, we tested whether fly larvae could use ethanol as an anti-parasite defense and whether its effectiveness would differ against the two wasp species. We found that fly larvae benefited from eating ethanol-containing food during exposure to *L*. *heterotoma*; we observed a two-fold decrease in parasitization intensity and a 24-fold increase in fly survival to adulthood. Although host ethanol consumption did not affect *L*. *boulardi* parasitization rates or intensities, it led to a modest increase in fly survival. Thus, ethanol conferred stronger protection against the generalist wasp than the specialist. We tested whether fly larvae can self-medicate by seeking ethanol-containing food after being attacked by wasps, but found no support for this hypothesis. We also allowed female flies to choose between control and ethanol-containing oviposition sites in the presence vs. absence of wasps and generally found significant preferences for ethanol regardless of wasp presence. Overall, our results suggest that *D*. *melanogaster* larvae obtain protection from certain parasitoid wasp species through their mothers’ innate oviposition preferences for ethanol-containing food sources.

## Introduction

Populations involved in antagonistic interactions often experience episodes of rapid, coupled evolutionary change. Host or prey populations undergo natural selection for new traits that confer enhanced resistance or tolerance against their enemies, and these new traits are repeatedly countered by adaptations in enemy populations. For example, plant defenses can increase in strength due to selection pressures from herbivores [1, 2] and parasites can evolve to avoid or impair host immune responses [3, 4]. However, we expect coevolutionary trajectories to differ between parasites that infect one or a few hosts (specialists) and parasites that infect a broader range of hosts (generalists). Populations of specialist parasites are distributed across narrower host ranges every generation, causing selection pressures to be more consistent across generations. Therefore, antagonistic coevolution is more likely between specialist parasites and their hosts, and specialist parasites will be more likely to counter host defenses than generalist parasites [5, 6].

Insect immune systems comprise physical, cellular, and humoral defenses [7]. Epithelial cells in the cuticle, gut, and tracheae serve as primary barriers to infection. They also produce antimicrobial peptides and reactive oxygen species that play important roles in local immune responses [8, 9]. Hemocytes are involved in several types of defenses, including clot formation, phagocytosis, nodulation, and encapsulation [10]. The fat body responds to systemic infections by producing antimicrobial peptides and secreting them into the hemolymph [11, 12]. However, recent studies have increasingly focused on alternative behavioral and symbiont-mediated defenses [13, 14]. For example, woolly bear caterpillars (*Grammia incorrupta*) that are parasitized by tachinid flies can improve their survival by increasing their ingestion of pyrrolizidine alkaloids [15]. In addition, protozoan-infected female monarch butterflies improve the fitness of their infected offspring by preferentially laying eggs on milkweed plants with high levels of cardenolides [16, 17]. Lastly, the mycophagous fly *Drosophila neotestacea* harbors a maternally transmitted mutualistic bacterium, *Spiroplasma*, which protects the fly against a sterilizing nematode parasite [18].

Larvae of the cosmopolitan fruit fly *Drosophila melanogaster* are commonly parasitized by endoparasitoid wasps in the genus *Leptopilina* (Hymenoptera: Cynipoidea, Figitidae), including *L*. *heterotoma*, a generalist that successfully parasitizes many *Drosophila* species, and *L*. *boulardi*, a specialist that is restricted to *D*. *melanogaster* and *D*. *simulans* across most of its geographical range [19–22]. Resistance against parasitoid wasps is critical to the long-term persistence of fruit fly populations, as natural rates of parasitism can exceed 90% [23]. Fly larvae can use a cellular immune response known as melanotic encapsulation to kill wasp eggs that have been laid inside them. However, venom released by wasps during oviposition can disrupt this response, allowing wasp larvae to consume and kill their host before emerging from the fly pupal case [22, 24, 25]. *D*. *melanogaster* cellular immune responses have often been found to be weak or completely ineffective against *Leptopilina*, as well as *Asobara* parasitoids [26–30]. Therefore, recent studies have investigated alternative behavioral defenses, such as avoidance of wasp-infested oviposition sites [28, 29] and use of ethanol for self-medication or kin medication [31, 32].

*D*. *melanogaster* larvae feed on yeasts that grow on fermenting fruits, in which ethanol concentrations can exceed 4% [33]. Some *D*. *melanogaster* populations thrive in and around wine cellars, using piles of discarded grape residues and barrel seepages as larval habitats, in which ethanol concentrations are often 7% or higher [34]. *D*. *melanogaster* has the highest adult ethanol tolerance among *Drosophila* species that use fermenting fruits as larval habitats, and adult ethanol tolerance is even higher in populations that breed in beer factories and wine cellars [35, 36]. This trait appears to have evolved in parallel between *D*. *melanogaster* and its parasitoids. Bouletreau and David [37] reported strong ethanol tolerance in six out of seven parasitoids of *Drosophila* larvae, with females having significantly higher tolerance than males, and hypothesized that this is an adaptation to avoid toxicity from the host larvae’s food sources during oviposition. Milan et al. [32] found that the specialist *L*. *boulardi* had significantly stronger tolerance to 6% and 8% ethanol than the generalist *L*. *heterotoma*. If *D*. *melanogaster* uses ethanol for defense against parasitoids, this difference in ethanol tolerance between parasitoid species may reflect the general prediction that specialist parasites are more likely to counter novel host defenses than generalist parasites. Here we follow this general prediction to test the following specific predictions: (i) *D*. *melanogaster* larvae consuming ethanol-containing food are better protected against generalist than specialist wasps; and (ii) *D*. *melanogaster* will actively use ethanol as a behavioral defense against generalist but not specialist wasps.

We tested the first prediction by growing *D*. *melanogaster* larvae in 0% or 6% ethanol food before, during, or after exposure to wasps, then measuring attack rates, parasitization intensities, and eclosion outcomes (fly survival, wasp survival, and death). With respect to our second prediction, *D*. *melanogaster* larvae could obtain ethanol in two ways: they might seek ethanol-containing food when foraging (self-medication) or female flies might preferentially oviposit in ethanol-containing food (kin medication) [31, 32]. We tested for both types of medication behavior by comparing preferences for 0% vs. 6% ethanol food between parasitized and unparasitized larvae, then comparing oviposition preferences for 0% vs. 6% ethanol food between wasp-exposed and unexposed female flies. We performed these experiments with the specialist *L*. *boulardi* and the generalist *L*. *heterotoma* to investigate the role of parasite–host specificity in the evolution of host defenses.

## Materials and methods

### Insect strains and maintenance

**Fig 1:**
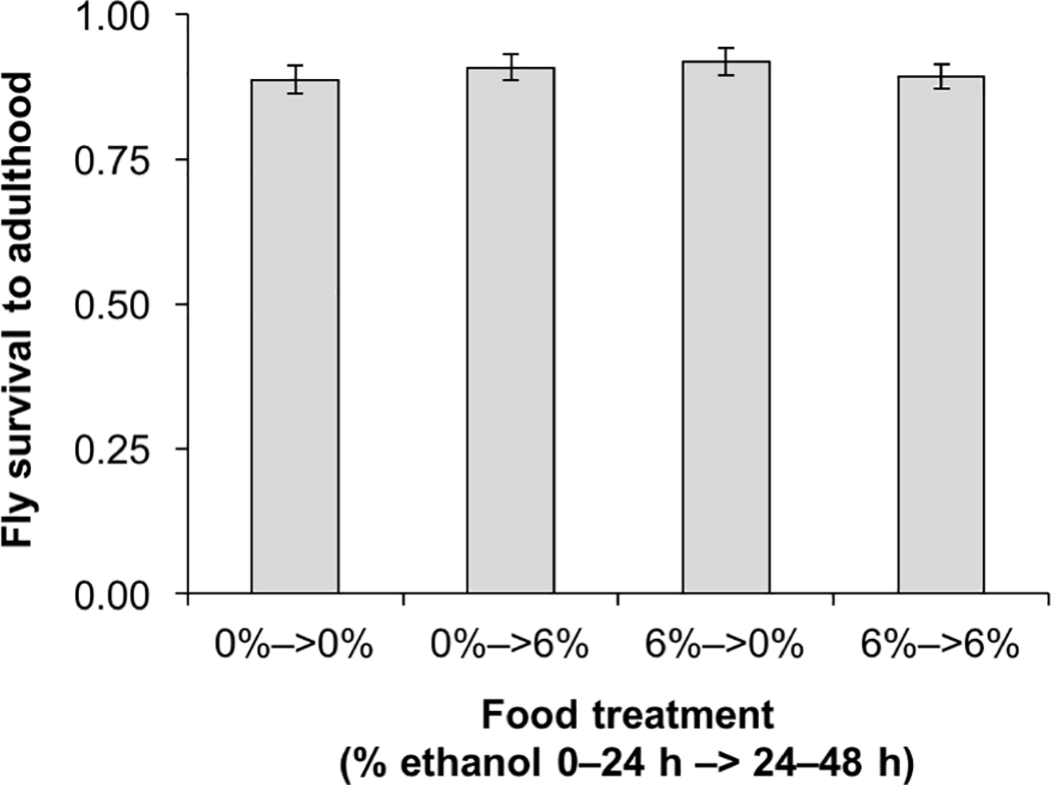
Effects of ethanol consumption on unparasitized larvae. Survival to adulthood of unparasitized second-instar *D*. *melanogaster* (strain *Oregon R*) larvae fed 0% or 6% ethanol food from 0–24 h, moved to new 0% or 6% ethanol food from 24–48 h, then transferred to standard fly food vials to complete development (4 replicates per treatment, 40 larvae per replicate, error bars: ± 1 SEM).

**Fig 2:**
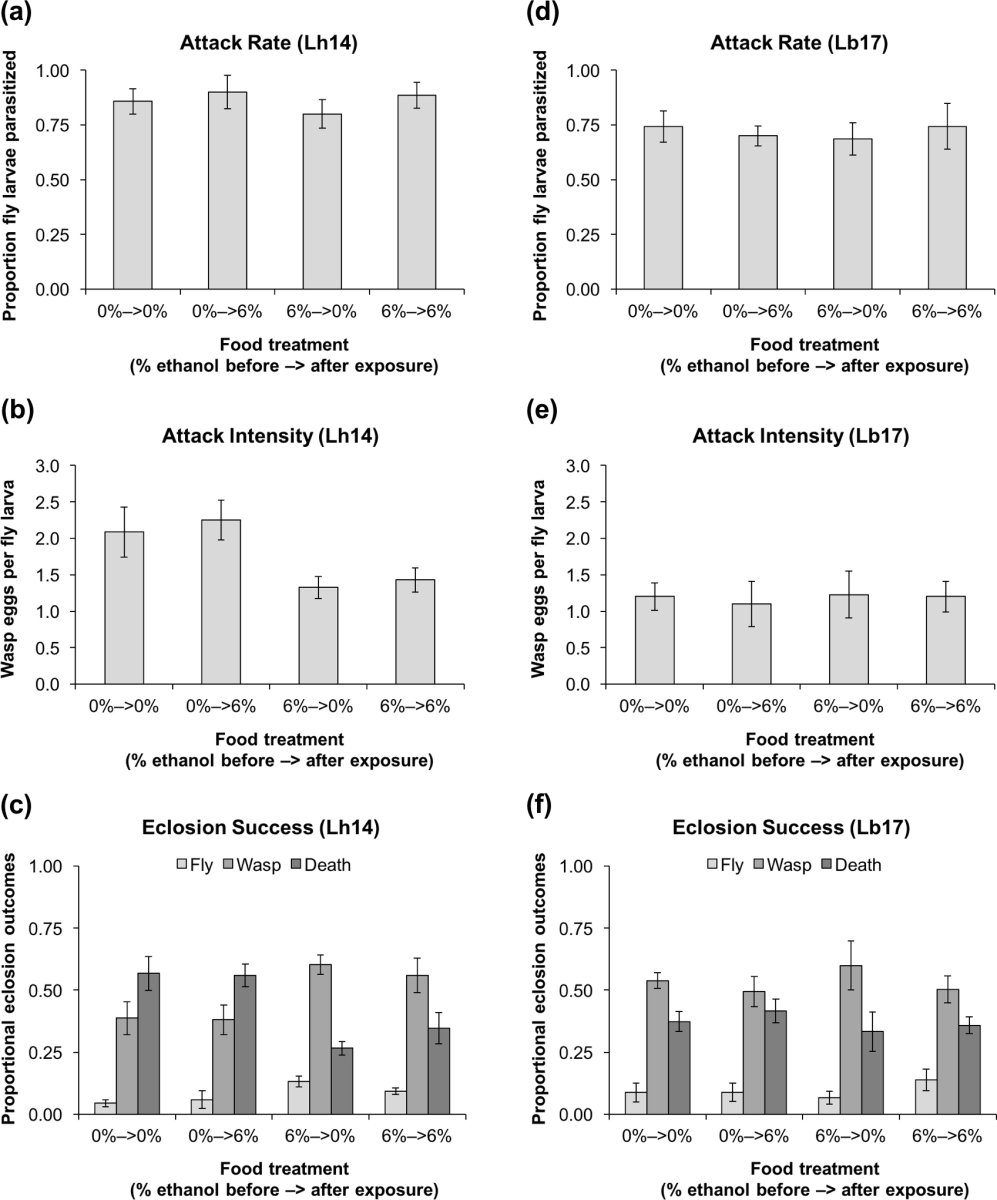
Effects of ethanol consumption before and after exposure to wasps. Proportion of second-instar *D*. *melanogaster* (strain *Oregon R*) larvae parasitized **(a,d)**, number of wasp eggs per fly larva **(b,e)**, and proportion of larvae that: (i) eclosed as flies, (ii) eclosed as wasps, or (iii) died **(c,f)**, when they were fed 0% or 6% ethanol food in the 24 hours before or after being exposed to *L*. *heterotoma* (Lh14) **(a,b,c)** or *L*. *boulardi* (Lb17) **(d,e,f)** (6–8 replicates per treatment, 5 larvae for dissections and ~30 for eclosions per replicate, error bars: ± 1 SEM).

**Fig 3:**
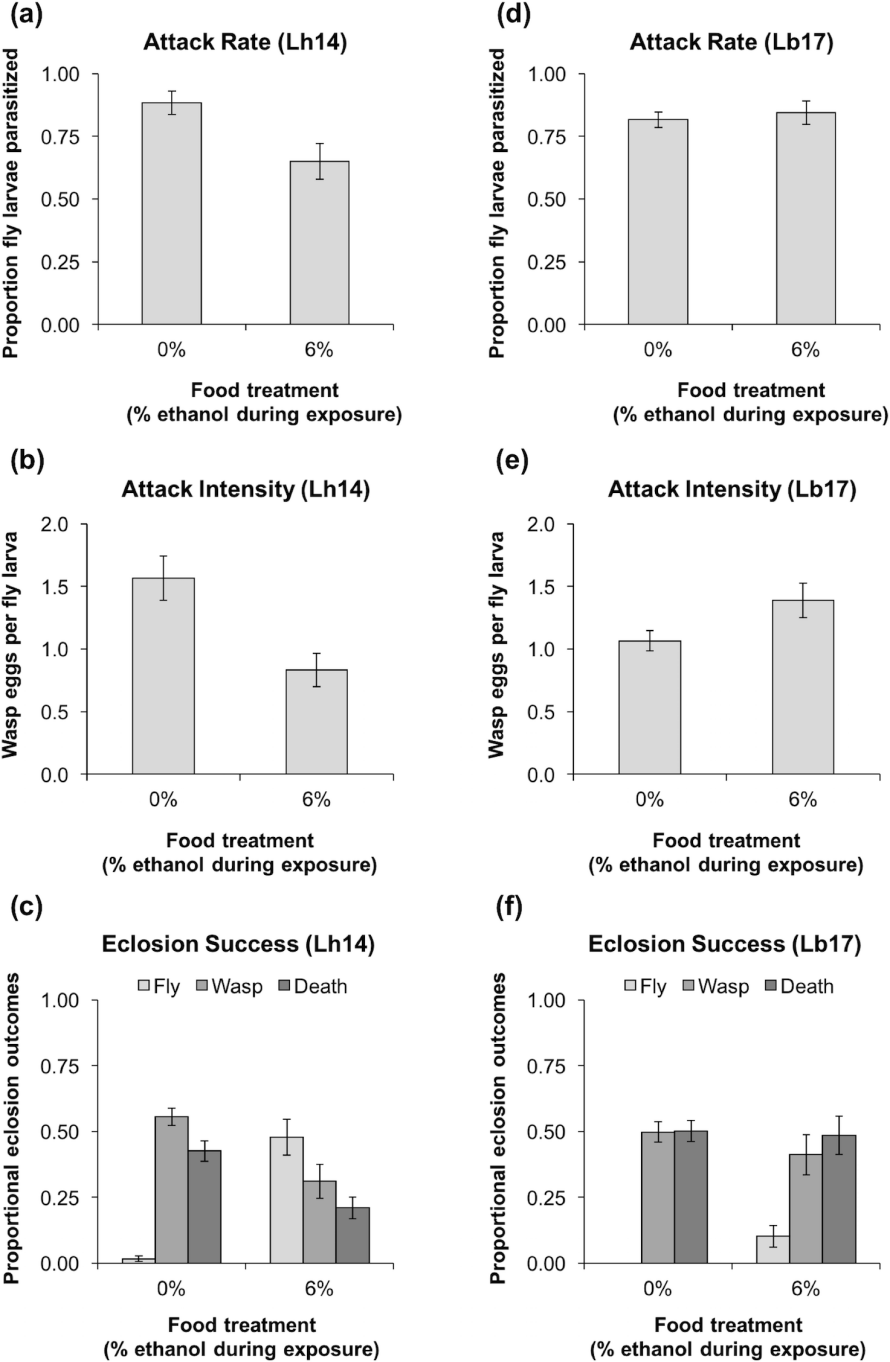
Effects of ethanol consumption during exposure to wasps. Proportion of third-instar *D*. *melanogaster* (strain *Oregon R*) larvae parasitized **(a,d)**, number of wasp eggs per fly larva **(b,e)**, and proportion of larvae that: (i) eclosed as flies, (ii) eclosed as wasps, or (iii) died **(c,f)**, when they were placed in 0% or 6% ethanol food and then immediately exposed to *L*. *heterotoma* (Lh14) **(a,b,c)** or *L*. *boulardi* (Lb17) **(d,e,f)** (6–7 replicates per treatment, 10 larvae for dissections and ~20 for eclosions per replicate, error bars: ± 1 SEM).

**Fig 4:**
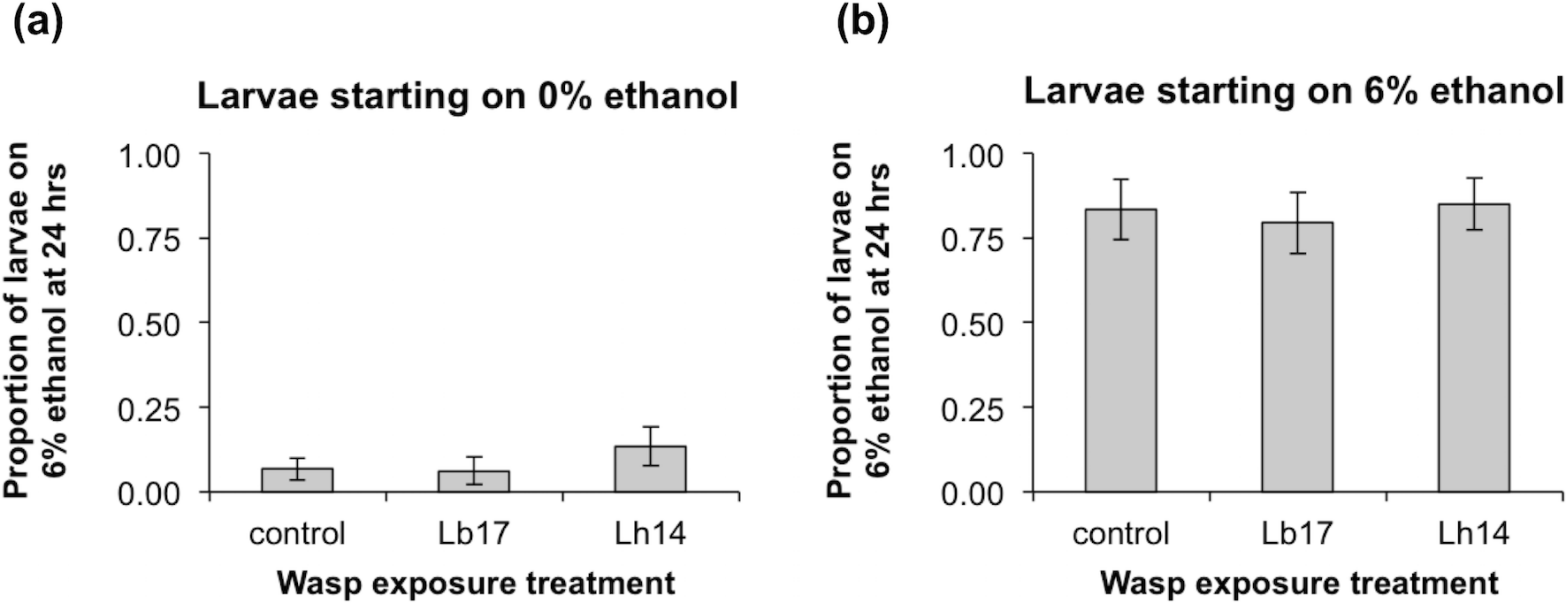
Larval ethanol food preference. Proportion of late second to early third-instar *D*. *melanogaster* (strain *Oregon R*) larvae in three wasp exposure treatments (unexposed controls, exposed to *L*. *boulardi* (Lb17), and exposed to *L*. *heterotoma* (Lh14)) that were on the 6% ethanol side at the end of a 24-hour choice experiment after starting on the 0% ethanol side **(a)** or the 6% ethanol side **(b)** of bisected Petri dishes (3 replicates per wasp exposure and starting side combination, ~70 larvae per replicate, error bars: ± 1 SEM).

**Fig 5:**
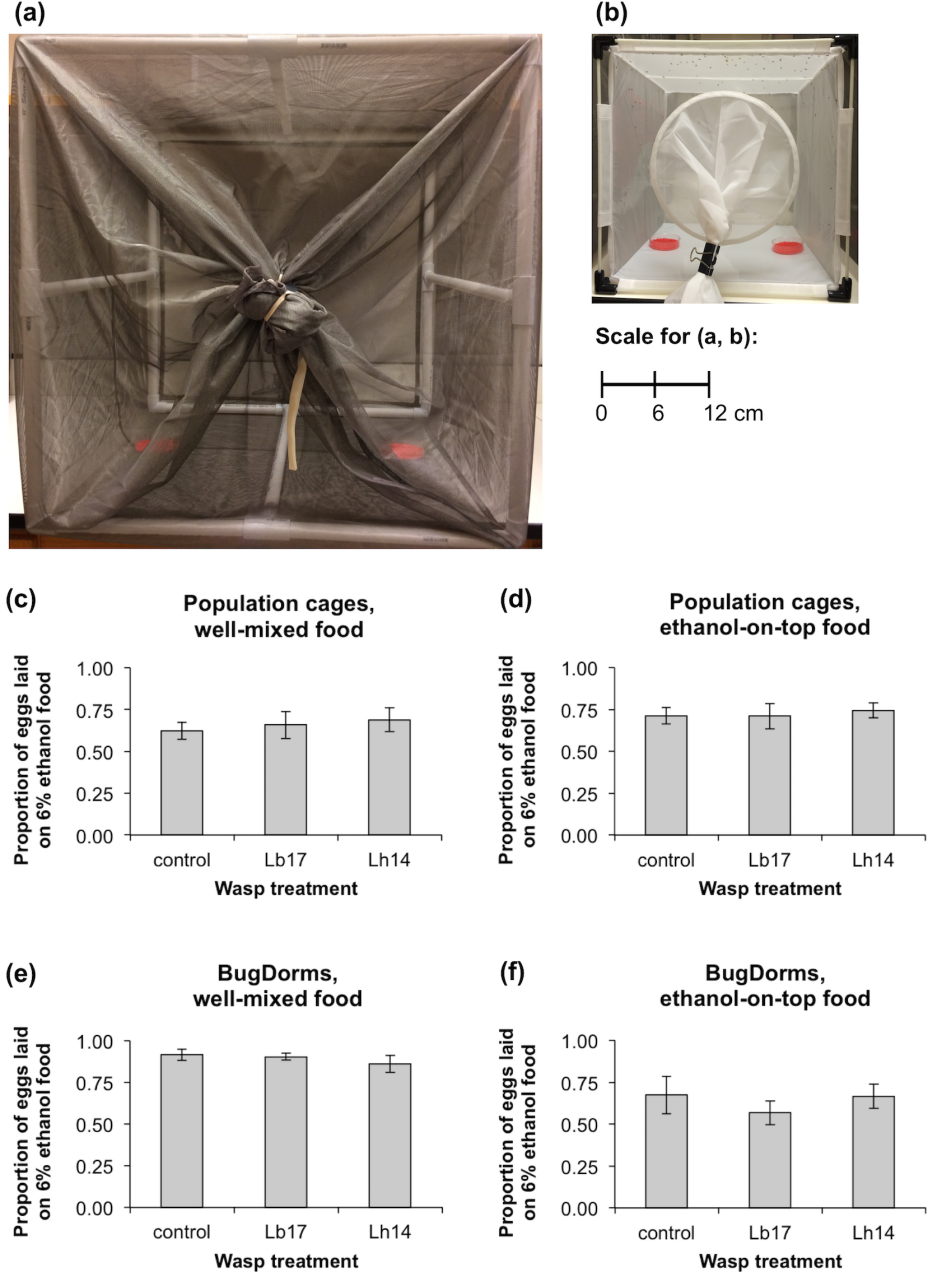
Adult ethanol oviposition preference. Proportion of eggs laid on 6% ethanol food when female *D*. *melanogaster* (strain *Canton S*) were allowed to choose between 0% and 6% ethanol food in the absence of wasps (control) or in the presence of female *L*. *boulardi* (Lb17) or *L*. *heterotoma* (Lh14). Two types of cages were used: population cages **(a,c,d)** and BugDorms **(b,e,f)**. Two methods of preparing the 6% ethanol dishes were compared: thoroughly mixing ethanol into the food **(c,e)** and pipetting 95% ethanol onto the surface of the food after adding the red 0% ethanol solution **(d,f)**. N = 6 per treatment in **(c,d)**; N = 6 (control) or 9 (Lb17 and Lh14) per treatment in **(e,f);** error bars: ± 1 SEM.

The *D*. *melanogaster* wild-type strains *Canton S* (strain 1) and *Oregon R* (strain 5) were obtained from the Bloomington Drosophila Stock Center. They were maintained in wide fly vials (#32–110, Genesee Scientific, San Diego, CA) on standard cornmeal-molasses-yeast medium [29]. For all experiments involving fly larvae, large groups of *Oregon R* flies (~200 adults) were placed in mesh-topped embryo collection chambers (#59–100, Genesee Scientific) with standard food in 60 mm diameter Petri dishes. Egglay dishes were collected and replaced every 24 h, then held for the appropriate amount of time (48–72 h from the midpoint of the egglay period) for larvae to reach second or third instar as required for each experiment. We used strain *Oregon R* in our larval fitness and migration experiments (Figs 1–4) to replicate a previous study [32] that used the same strain for similar experiments. We used strain *Canton S* in our adult oviposition preference experiments (Fig 5) to replicate a previous study [31] that used the same strain for similar experiments. Specifically, we used the same *Oregon R* and *Canton S* stocks that were used in the previous studies [31, 32]; these stocks were maintained at Emory University through December 2016.

The endoparasitoid wasps *Leptopilina boulardi* (strain Lb17) and *L*. *heterotoma* (strain Lh14) are inbred strains generated from single females collected in Winters, CA in 2002 [22]. To maintain wasp stocks, *Canton S* flies laid eggs in food vials for three days, then the flies were removed and ~10 mated female wasps were added. Newly emerged male and female wasps were placed in food vials with one-half rolled Kimwipe pushed into the center of the food and fed by adding a 50–50 honey-water solution to the cellulose acetate Flug (#49–101, Genesee Scientific). They were held for 3–7 days before each experiment to ensure the female wasps had mated. Insect stocks were kept on the lab bench under ambient temperature, humidity, and light conditions. Environmental conditions measured during our experiments generally ranged from 23–25°C and 30–34% relative humidity, although an HVAC failure led to different conditions during one day of an oviposition preference trial, as explained later. Overhead lights were usually on during work hours and off at night. Most experiments also took place under these ambient light conditions, except for the larval food preference and adult oviposition preference experiments, which used a 15 h light: 9 h dark cycle.

### Recipes for colored ethanol solutions

All experiments involved preparing fly food at 0% and 6% ethanol, which are within the range of ethanol concentrations experienced by *D*. *melanogaster* larvae in their natural food sources [33, 34]. Instant Drosophila medium (Formula 4–24 plain, Carolina Biological Supply, Burlington, NC) was mixed with the appropriate volume of a colored 0% or 6% ethanol solution. The stock 0% ethanol solution was made in 500 mL batches with 495 mL reverse osmosis water and 5 mL red food coloring (McCormick, Sparks, MD). This made it easier to see fly eggs and larvae in the instant food. Fresh batches of 6% ethanol solution were made immediately before each experiment by mixing appropriate amounts of the stock solution and 100% ethanol (Decon Laboratories, King of Prussia, PA). For example, to make 15 Petri dishes with 1 mL of liquid each, 14.1 mL stock was mixed with 0.9 mL 100% ethanol, then pipetted onto the instant food in 1 mL aliquots. The food mixing method described above was used for all types of experiments, but in the section “Adult ethanol oviposition preference,” we describe an additional mixing method that was only tested in those experiments. Therefore, in that section we refer to the standard method as “well-mixed food” and the new method as “ethanol-on-top food.”

### Effects of ethanol consumption on unparasitized larvae

Fly food was prepared in 35 mm diameter Petri dishes by mixing 0.25 g instant Drosophila medium and ~10 granules of live baker’s yeast (Fleischmann’s, AB Mauri, St. Louis, MO) with 1 mL red 0% or 6% ethanol solution. Sets of 40 second-instar *Oregon R* larvae were placed in 0% or 6% ethanol food dishes for 24 h, transferred to new dishes with 0% or 6% ethanol food for another 24 h, then placed in cornmeal-molasses-yeast food vials to complete development. This generated four treatments: 0%–>0%, 0%–>6%, 6%–>0%, and 6%–>6%. Surviving adult flies were counted approximately 10 days later. The effect of food treatment on proportional fly survival was assessed using a generalized linear model (GLM) with quasi-binomial error distribution and logit link function. All statistical analyses were performed in R version 3.2.3 [38].

### Effects of ethanol consumption before and after exposure to wasps

Fly food was prepared in 35 mm diameter Petri dishes by mixing 0.25 g instant Drosophila medium and ~10 granules of live baker’s yeast with 1 mL red 0% or 6% ethanol solution. Sets of 40 second-instar *Oregon R* larvae were placed in 0% or 6% ethanol food dishes, where they fed for 24 h before being exposed to 10 female wasps (Lb17 or Lh14) for 2 h. They were then moved from their original dishes to new dishes with 0% or 6% ethanol food, where they fed for 24 h. This generated four treatments: 0%–>0%, 0%–>6%, 6%–>0%, and 6%–>6%. Five larvae from each dish were dissected to count the number of parasitoid eggs laid inside them. The remaining larvae were transferred to cornmeal-molasses-yeast food vials to develop and eclose. Final eclosion success was calculated from the number of larvae transferred, and most vials had fewer than 35 larvae because some died or could not be found. Surviving adult flies and wasps were counted approximately one week and four weeks later, respectively.

The effects of food given before exposure to wasps on the proportion of dissected fly larvae that had been parasitized were assessed using GLMs with quasi-binomial error distributions and logit link functions. The effects of food given before exposure to wasps on the number of wasp eggs laid in each dissected fly larva were assessed using GLMs with quasi-Poisson error distributions and log link functions. The effects of food given (i) before exposure to wasps and (ii) after exposure to wasps on proportional eclosion outcomes (fly survival, wasp survival, and death of both fly and wasp) were assessed using GLMs with quasi-binomial error distributions and logit link functions.

### Effects of ethanol consumption during exposure to wasps

Fly food was prepared in 35 mm diameter Petri dishes by mixing 0.25 g instant Drosophila medium and ~10 granules of live baker’s yeast with 1 mL red 0% or 6% ethanol solution. Sets of 30 early third-instar *Oregon R* larvae were placed in 0% or 6% ethanol food dishes and immediately exposed to 10 female wasps (Lb17 or Lh14) for 2 h. The larvae fed in their dishes for 12 h after the wasps were removed, then 10 larvae from each dish were dissected to count the number of parasitoid eggs laid inside them. The remaining larvae were transferred to cornmeal-molasses-yeast food vials to develop and eclose. Final eclosion success was calculated from the number of larvae transferred, and most vials had fewer than 20 larvae because some died or could not be found. Surviving adult flies and wasps were counted approximately one week and four weeks later, respectively.

The effects of food treatment on the proportion of dissected fly larvae that had been parasitized were assessed using GLMs with quasi-binomial error distributions and logit link functions. The effects of food treatment on the number of wasp eggs laid in each dissected fly larva were assessed using GLMs with quasi-Poisson error distributions and log link functions. The effects of food treatment on proportional eclosion outcomes (fly survival, wasp survival, and death of both fly and wasp) were assessed using GLMs with quasi-binomial error distributions and logit link functions.

### Larval ethanol food preference

Nine sets of 150 late second to early third-instar *Oregon R* larvae were placed in 60 mm diameter Petri dishes with cornmeal-molasses-yeast food. The larvae were exposed to 20 female wasps (Lb17 or Lh14) for 3 h or left as unexposed controls (3 dishes per treatment). Larval choice environments were then set up in bisected 100 mm diameter Petri dishes (Thermo Fisher Scientific, Waltham, MA) with 1 g instant Drosophila medium and 10–20 granules of live baker’s yeast on each side, plus 4 mL of red liquid: 0% ethanol on one side and 6% ethanol on the other side. Larvae from each 60 mm dish were divided equally between two 100 mm choice dishes, one with the larvae starting on 0% ethanol and one with the larvae starting on 6% ethanol. Overall, there were three wasp exposure treatments (control, Lb17, and Lh14) and two starting side treatments (0% and 6%), with three replicates per combination and ~70 larvae per replicate. The larvae were given 24 h to move freely within their bisected food dishes. After this choice period, larvae on the 0% and 6% sides of each dish were counted as they were removed from the food. Ten larvae from each original wasp exposure dish (30 total per wasp species) were dissected to count the number of parasitoid eggs laid inside them. Ambient lab conditions ranged from 24–25°C and 30–32% relative humidity during this trial and a 15 h light: 9 h dark cycle was used. The effects of wasp treatment on the proportion of larvae that were on the 6% ethanol side following the 24 h choice period were assessed using GLMs with quasi-binomial error distributions and logit link functions. The 0% and 6% ethanol starting side treatments were analyzed separately.

### Adult ethanol oviposition preference

We conducted oviposition preference experiments in which groups of female *Canton S* flies were allowed to choose between control (0% ethanol) and 6% ethanol food dishes in cages with or without female parasitoid wasps. We used two types of cages and tested two different methods of mixing the fly food. Every trial involved collecting data from a group of cages at multiple time points, so we placed the dishes on opposite sides of the cages and switched the position of the 6% ethanol dish between time points to ensure that we were measuring ethanol preference rather than side bias.

We ran one set of experiments in 60 x 60 x 60 cm population cages constructed from white PVC pipe covered with coarse brown/green mesh. We observed some parasitoid wasps escaping through the mesh, indicating that these cages are not ideal for experiments with wasps. However, these cages were used in a previous study [31], and our goal was to replicate their setup. Groups of 300 female flies (3–6 days old) were released into cages with 50 female wasps (Lb17 or Lh14) or without wasps (control) and given two food dishes, one with 0% ethanol and one with 6% ethanol, spaced approximately 40 cm apart between the centers of the dishes. These dishes were replaced 24 h later and eggs laid in each dish were counted following both choice periods (0–24 h and 24–48 h). In our first experiment, we prepared the fly food by mixing 4 g instant Drosophila medium with 16 mL red 0% or 6% ethanol solution in 100 mm diameter Petri dishes (well-mixed food). In our second experiment, we instead prepared the 6% ethanol dishes by adding 15 mL red 0% ethanol solution to 4 g instant Drosophila medium, then slowly dispensing 1 mL 95% ethanol across the surface of the food using a micropipette (ethanol-on-top food), following the protocol used in [31] and [39]. Ambient lab conditions ranged from 23–24°C and 31–34% relative humidity during these trials and a 15 h light: 9 h dark cycle was used. The effects of choice period, wasp treatment, and their interaction on the proportion of eggs laid on 6% ethanol food were assessed using GLMs with quasi-binomial error distributions and logit link functions.

To address the problem of wasps escaping from the population cages and determine whether our conclusions were robust to changes in the experimental setup, we ran another set of experiments in 30 x 30 x 30 cm BugDorm-43030 insect rearing cages (MegaView Science, Taichung City, Taiwan). These cages have fine mesh that prevents wasps from escaping. Groups of 100 female flies (3–6 days old) were released into cages with 20 female wasps (Lb17 or Lh14) or without wasps (control) and given two food dishes, one with 0% ethanol and one with 6% ethanol, spaced approximately 20 cm apart between the centers of the dishes. These dishes were replaced 24 and 48 h after the start of the experiment. Eggs laid in each dish were counted following these three choice periods (0–24 h, 24–48 h, and 48–72 h). In our first experiment, we prepared the fly food by mixing 2 g instant Drosophila medium with 8 mL red 0% or 6% ethanol solution in 60 mm diameter Petri dishes (well-mixed food). In our second experiment, we instead prepared the 6% ethanol dishes by adding 7.5 mL red 0% ethanol solution to 2 g instant Drosophila medium, then slowly dispensing 0.5 mL 95% ethanol across the surface of the food using a micropipette (ethanol-on-top food). Ambient lab conditions generally ranged from 23–25°C and 30–32% relative humidity during these trials and a 15 h light: 9 h dark cycle was used. Due to a temporary HVAC failure, ambient conditions were 20–22°C and 37–44% relative humidity during one day of one trial, but this did not have any obvious effect on our results. The effects of choice period, wasp treatment, and their interaction on the proportion of eggs laid on 6% ethanol food were assessed using GLMs with quasi-binomial error distributions and logit link functions.

## Results

### Effects of ethanol consumption on unparasitized larvae

To investigate the possible fitness costs of consuming ethanol when unparasitized, we fed *D*. *melanogaster* (strain *Oregon R*) larvae 0% or 6% ethanol food in the 48 hours after reaching second instar. There was no difference in survival to adulthood across food treatments (Fig 1; *F*_*3*,*11*_ = 0.39, *P* = 0.76), suggesting that consuming 6% ethanol food during second and third instar is not costly to fly fitness.

### Effects of ethanol consumption before and after exposure to wasps

Next, we investigated the effects of consuming ethanol in the 24 hours before and after exposure to wasps. Second-instar *D*. *melanogaster* (strain *Oregon R*) larvae grown in 6% ethanol food for 24 hours before exposure to the generalist *L*. *heterotoma* were not less likely to be parasitized (Fig 2A; *F*_*1*,*28*_ = 0.37, *P* = 0.55), but had significantly fewer parasitoid eggs laid inside them (Fig 2B; *F*_*1*,*28*_ = 12.7, *P* = 0.001). This led to significantly higher fly survival (Fig 2C; *F*_*1*,*28*_ = 5.8, *P* = 0.02) and wasp survival (*F*_*1*,*28*_ = 11.2, *P* = 0.002), along with lower death rates (*F*_*1*,*28*_ = 24.2, *P* < 0.0001).

None of these effects were observed with the specialist wasp. Fly larvae grown in 6% ethanol food for 24 hours before exposure to *L*. *boulardi* did not experience lower parasitization rates (Fig 2D; *F*_*1*,*25*_ = 0.013, *P* = 0.91) or intensities (Fig 2E; *F*_*1*,*25*_ = 0.058, *P* = 0.81) and there were no effects on any eclosion outcome (Fig 2F; fly: *F*_*1*,*25*_ = 0.16, *P* = 0.69; wasp: *F*_*1*,*25*_ = 0.20, *P* = 0.66; death: *F*_*1*,*25*_ = 0.73, *P* = 0.40).

Consuming 6% ethanol in the 24 hours after exposure to wasps did not have a significant effect on any eclosion outcome when larvae were exposed to *L*. *heterotoma* (Fig 2C; fly: *F*_*1*,*28*_ = 0.49, *P* = 0.49; wasp: *F*_*1*,*28*_ = 0.32, *P* = 0.58; death: *F*_*1*,*28*_ = 0.66, *P* = 0.43) or *L*. *boulardi* (Fig 2F; fly: *F*_*1*,*25*_ = 1.2, *P* = 0.29; wasp: *F*_*1*,*25*_ = 1.1, *P* = 0.30; death: *F*_*1*,*25*_ = 0.31, *P* = 0.58). These results suggest that ethanol can protect fly larvae against the generalist *L*. *heterotoma* but not the specialist *L*. *boulardi*, and only when it is consumed before exposure to wasps.

### Effects of ethanol consumption during exposure to wasps

Ethanol is continuously produced as yeasts grow on fermenting fruits, whereas ethanol concentrations decrease over time in artificial lab medium due to evaporation [33]. In our previous experiments, fly larvae were placed in Petri dishes with 6% ethanol food 24 hours before being exposed to wasps. This may have led us to under-estimate the effects of ethanol on wasp oviposition behavior. In the following experiments, we attempted to maximize these effects by placing fly larvae in Petri dishes with 6% ethanol food, then immediately adding wasps to the dishes.

Third-instar *D*. *melanogaster* (strain *Oregon R*) larvae that were placed in 6% ethanol food and then immediately exposed to the generalist *L*. *heterotoma* were attacked significantly less often (Fig 3A; *F*_*1*,*10*_ = 7.0, *P* = 0.024) and had significantly fewer parasitoid eggs laid inside them (Fig 3B; *F*_*1*,*10*_ = 11.1, *P* = 0.008). This led to significantly higher fly survival (Fig 3C; *F*_*1*,*10*_ = 54.2, *P* < 0.0001), whereas wasp survival and death were significantly reduced (wasp: *F*_*1*,*10*_ = 9.98, *P* = 0.010; death: *F*_*1*,*10*_ = 12.4, *P* = 0.006).

When we performed the same experiment with the specialist *L*. *boulardi*, fly larvae did not experience lower parasitization rates (Fig 3D; *F*_*1*,*11*_ = 0.20, *P* = 0.66) or intensities (Fig 3E; *F*_*1*,*11*_ = 1.3, *P* = 0.28). However, fly survival was significantly higher (Fig 3F; *F*_*1*,*11*_ = 9.9, *P* = 0.009) despite no effects on wasp survival (*F*_*1*,*11*_ = 0.54, *P* = 0.48) or death (*F*_*1*,*11*_ = 0.06, *P* = 0.81).

These results provide additional evidence that ethanol confers protection against the generalist *L*. *heterotoma*. Consuming ethanol food in the 24 hours before wasp exposure led to a two-fold increase in fly survival (Fig 2C), compared to a 24-fold increase in fly survival when fly larvae were placed in ethanol food and then immediately exposed to wasps (Fig 3C). Ethanol did not decrease *L*. *boulardi* attack rates or intensities in either experiment (Fig 2D and 2E; Fig 3D and 3E) but led to increased fly survival in the second experiment (Fig 3F), suggesting that ethanol provides limited protection against the specialist wasp. In all of our fitness experiments, wasps were allowed to attack fly larvae in ethanol-containing food for 2-hour periods. No mortality was observed among the attacking wasps, suggesting that our exposure scenario led to altered wasp behaviors but not differential wasp mortality.

### Larval ethanol food preference

To determine whether fly larvae would preferentially seek ethanol food and whether this behavior would change following wasp attack, we gave *D*. *melanogaster* (strain *Oregon R*) larvae 24 hours to freely migrate in bisected Petri dishes with 0% and 6% ethanol food. Compared to unexposed control larvae, wasp-exposed larvae did not show increased migration from the 0% ethanol side to the 6% ethanol side (Fig 4A; *F*_*2*,*6*_ = 0.82, *P* = 0.48). Exposure to wasps also had no effect on the propensity of fly larvae to stay in 6% ethanol food when they started there (Fig 4B; *F*_*2*,*6*_ = 0.09, *P* = 0.91). The overall tendency, regardless of wasp exposure treatment and starting side, was to stay on the starting side (*t*_*17*_ = 8.1, *P* < 0.0001). Lack of wasp exposure effects cannot be explained by low attack rates: 83.3 ± 0.03% and 96.7 ± 0.03% of larvae exposed to *L*. *boulardi* and *L*. *heterotoma* (respectively) contained wasp eggs. Neither unparasitized nor parasitized *D*. *melanogaster* larvae preferentially migrated towards 6% ethanol food. Thus, we found no evidence for self-medication behavior.

The results from a similar experiment carried out previously with less control of temperature, humidity and light cycle were qualitatively equivalent to the results described here (S1 Appendix).

### Adult ethanol oviposition preference

To determine whether parasitoid wasps affect female flies’ oviposition preference for ethanol, we placed female *D*. *melanogaster* (strain *Canton S*) in cages with or without female wasps and allowed the flies to choose between 0% and 6% ethanol oviposition sites. We investigated the generality of this behavior by using two types of cages and two different methods of mixing the fly food.

Our first set of experiments was conducted in 60 x 60 x 60 cm population cages (Fig 5A). When the food was prepared by aliquoting 0% or 6% ethanol solution onto the instant Drosophila medium (well-mixed food), flies showed a significant preference for 6% ethanol oviposition sites (Fig 5C; *t*_*17*_ = 3.0, *P* = 0.008) and there was no effect of wasp treatment (*F*_*2*,*15*_ = 0.23, *P* = 0.80). Similarly, when 1 mL 95% ethanol was pipetted onto the surface of the food after adding 15 mL red 0% ethanol solution (ethanol-on-top food), flies showed a significant preference for 6% ethanol oviposition sites (Fig 5D; *t*_*17*_ = 5.9, *P* < 0.0001) and there was no effect of wasp treatment (*F*_*2*,*15*_ = 0.24, *P* = 0.79). For both experiments, data were pooled across the two choice periods (0–24 and 24–48 hours) because there was no effect of choice period on oviposition preference (*F*_*1*,*14*_ < 1.7, *P* > 0.22) and no significant interaction between choice period and wasp treatment (*F*_*2*,*12*_ < 0.68, *P* > 0.53).

The results from a similar experiment carried out previously with less control of temperature, humidity and light cycle were qualitatively equivalent to the results described here, except that the trials with well-mixed food did not reveal a significant preference for 6% ethanol oviposition sites (S1 Appendix).

Our second set of experiments was conducted in 30 x 30 x 30 cm BugDorm-43030 cages (Fig 5B). In the experiment with well-mixed food, flies showed a significant preference for 6% ethanol oviposition sites (Fig 5E; *t*_*23*_ = 9.6, *P* < 0.0001) and there was no effect of wasp treatment (*F*_*2*,*21*_ = 0.19, *P* = 0.83). However, in the experiment with ethanol-on-top food, flies did not show a significant preference for either oviposition site (Fig 5F; *t*_*23*_ = 1.5, *P* = 0.16) and there was no effect of wasp treatment (*F*_*2*,*21*_ = 1.4, *P* = 0.29). Both BugDorm experiments showed significant effects of choice period on oviposition preference (*F*_*2*,*19*_ > 6.6, *P* < 0.009). Ethanol preference was weakest from 48–72 hours with well-mixed food and weakest from 0–24 hours with ethanol-on-top food. However, data were pooled across the three choice periods for both experiments because there was no significant interaction between choice period and wasp treatment (*F*_*4*,*15*_ < 1.9, *P* > 0.17). Female *D*. *melanogaster* preferentially laid eggs on 6% ethanol food in the majority of our experiments and the presence or absence of female wasps never had a significant effect on this behavior.

## Discussion

We investigated whether *D*. *melanogaster* larvae can use ethanol for protection against parasitoid wasps. We also tested whether ethanol use is driven by choices at the larval or adult stages and whether its effectiveness differs against generalist vs. specialist wasps. We found that unparasitized second-instar fly larvae were equally likely to survive to adulthood when they consumed 0% or 6% ethanol food (Fig 1), suggesting that ethanol consumption may not carry fitness costs in the absence of parasitoids. A previous study that also used the *D*. *melanogaster* wild-type strain *Oregon R* similarly found that 4% to 8% ethanol food did not affect fly survival [32], although studies using different wild-type strains have found reduced fly survival at ethanol concentrations above 3% [40, 41]. Second-instar larvae that consumed 6% ethanol food in the 24 hours before exposure to wasps were not less likely to be parasitized by the generalist *L*. *heterotoma* or the specialist *L*. *boulardi*. Consuming ethanol before exposure to *L*. *heterotoma* led to lower parasitization intensities and higher fly survival, but food consumed in the 24 hours after exposure had no effect on fly survival against either wasp (Fig 2). When we placed third-instar larvae in 6% ethanol food and immediately exposed them to wasps, parasitization rates and intensities were only reduced for *L*. *heterotoma*, although fly survival was higher against both wasp species (Fig 3). However, significantly increased fly survival was only coupled with significantly reduced wasp survival in one scenario, when fly larvae were placed in 6% ethanol food and then immediately exposed to the generalist *L*. *heterotoma* (Fig 3C). When ethanol was administered 24 hours before exposure, fly and wasp survival were both significantly increased (Fig 2C). This suggests that constant access to ethanol is highly beneficial to the long-term persistence of *D*. *melanogaster* populations threatened by the generalist wasp. Overall, we found that ethanol provides effective protection against the generalist *L*. *heterotoma* but limited protection against the specialist *L*. *boulardi*, similar to the findings of Milan et al. [32].

Previous laboratory choice experiments showed that unparasitized *D*. *melanogaster* larvae prefer 6% ethanol food to 0% ethanol food [42, 43] and that larvae parasitized by *L*. *boulardi* and *L*. *heterotoma* have even stronger preferences for 6% ethanol food [32]. However, the results of our fitness experiments (Figs 2 and 3) suggest that preferential consumption of ethanol would not be equally effective against the generalist and the specialist wasp. In our food choice experiments, *D*. *melanogaster* larvae did not migrate towards 6% ethanol food even after they had been parasitized (Fig 4). We found no evidence that fly larvae self-medicate with ethanol to cure wasp infections. Therefore, our results categorically differ from those of Milan et al. [32], even though we followed similar protocols and used the same type of fly food. Instead, our results suggest that the ability of larvae to exploit ethanol for anti-wasp defense might be primarily dictated by adult oviposition preferences rather than larval food preferences. This seems likely because some fruits that serve as natural habitats for *D*. *melanogaster* have little or no ethanol [33] and flying adults are better able to move between fruits than crawling larvae.

Female flies could preferentially lay their eggs in ethanol-containing food to protect their offspring from future wasp parasitization, an example of kin medication behavior [44]. *D*. *melanogaster* has evolved high ethanol tolerance [35] and exploits ethanol-rich food sources avoided by its sister species, *D*. *simulans* [34, 41]. This suggests that female *D*. *melanogaster* will prefer ethanol-laden oviposition sites regardless of whether they encounter wasps, and multiple studies have reported innate ethanol preference [45–48]. However, other studies have found that ethanol oviposition preference is significantly increased following exposure to wasps [31, 39]. We found significant preferences for 6% ethanol oviposition sites in three out of four experiments and none of our experiments showed an effect of wasp exposure (Fig 5). Our results support innate ethanol preference in *D*. *melanogaster*, not wasp-induced ethanol-seeking behavior as reported in [31] and [39], even though we repeated the oviposition choice experiment described in [31] and tested the ethanol-on-top food mixing method described in [39]. Overall, we found that fly larvae were significantly more likely to survive exposure to wasps when they consumed ethanol food (Figs 2 and 3) but did not seek out ethanol food even after being attacked by wasps (Fig 4). Taken together, these results strongly suggest that innate oviposition preference for ethanol (Fig 5) protects fly offspring against *L*. *boulardi*, *L*. *heterotoma*, and probably other parasitoid wasp species that have lower ethanol tolerance than *D*. *melanogaster* larvae.

Ethanol oviposition preference is a labile behavior in laboratory assays [46] and studies have often reported conflicting results [47]. We conducted choice experiments in two types of cages with different insect densities and food recipes to test the generality of our conclusions. Our result that female *D*. *melanogaster* generally prefer to oviposit in high-ethanol food is consistent with some studies [45–48] but inconsistent with others [31, 39], which found that wasp-exposed, but not unexposed, flies preferred to oviposit in food with ethanol. One possible explanation is that different fly genotypes may have different oviposition preferences. However, our results are consistent with previous studies that used different *D*. *melanogaster* strains [45–48] but inconsistent with previous studies that also used the strain *Canton S* [31, 39]. Alternatively, a recent study showed that the distance between oviposition sites can strongly affect ethanol oviposition preferences in *D*. *melanogaster* and other fly species [49]. However, the oviposition sites in our population cage experiments were approximately 40 cm apart (between the centers of the dishes), very similar to the setups used in [31] and [39], and our results were consistent with other studies that provided multiple control and ethanol-containing sites with varying distances between them [46–48] or adjacent, binary options [45].

Another phenomenon that may produce inconsistencies between choice experiments is that artificial food sources start with a fixed amount of ethanol that continuously evaporates. This happens slowly in plugged containers and very quickly in open Petri dishes [33]. Therefore, ethanol concentrations should remain more constant when choice periods are shorter and ventilation is reduced in the experimental containers. Studies using choice periods of 3 hours or shorter have reported significant ethanol preferences [45, 46], whereas studies using 16-hour choice periods have produced mixed results [41, 47, 48, 50]. Two studies that ran choice assays in small bottles instead of large cages both found significant ethanol preferences despite using different choice periods: 3 hours [45] and 16 hours [47]. Limiting the effects of ethanol evaporation may improve the reproducibility of choice experiments, but the relevance of such experiments to our understanding of natural fly behaviors remains unclear. Laboratory setups may not accurately reflect spatiotemporal variation in ethanol concentrations across naturally fermenting fruits, and other environmental factors can contribute to fly oviposition decisions and resistance against parasitoid wasps.

Experiments using artificial food sources are not sufficient to demonstrate that medication behaviors are relevant in an organism’s natural environment [44]. To improve the ecological relevance of choice experiments and perhaps resolve inconsistencies across studies, future experiments should better approximate the natural oviposition environments of flies and wasps. For example, flies could choose between fruits with or without yeast in the presence or absence of wasps, using yeast species that differ in their fermentation propensities. Female *D*. *melanogaster* tend to prefer *Saccharomyces* species that opt for fermentation even in aerobic conditions (the Crabtree effect) [51]. These yeasts dominate fruit niches by producing ethanol and heat that other species cannot tolerate [52]. However, ethanol is not the only volatile compound involved in attracting female flies; the fermentation headspace of *Saccharomyces cerevisiae* also includes acetic acid, acetoin, 2-phenyl ethanol, and 3-methyl-1-butanol [53]. Although *S*. *cerevisiae* is often used in studies involving fruit flies and yeast, it is not commonly associated with *Drosophila* in nature, so future studies may benefit from testing yeast strains that have been isolated from wild-caught flies [54]. Understanding natural yeast communities seems especially important because *D*. *melanogaster* engages in niche construction. Adult flies vector yeasts between fruits and larvae assemble them into communities with predictable compositions and densities [55]. Therefore, choice experiments that only test the effects of ethanol ignore several potentially important aspects of tri-trophic interactions between flies, fruits, and yeasts.

Furthermore, substances besides ethanol may influence the ability of *D*. *melanogaster* offspring to avoid or resist parasitoid wasps. Gibson et al. [33] found that apples, citrus fruits, melons, and tomatoes that served as natural hosts for *D*. *melanogaster* larvae contained little or no ethanol. However, citrus fruits might offer a different type of protection against wasps: volatile terpenes in their rinds attract ovipositing *D*. *melanogaster* but deter foraging *L*. *boulardi* [56]. Besides producing ethanol, yeasts provide essential nutrients that can affect the cellular immune responses of fly larvae. Anagnostou et al. [57] reported that *D*. *melanogaster* larvae had significantly different development times and melanotic encapsulation success against the braconid wasp *Asobara tabida* when they consumed different yeast species. In two-choice assays, infected larvae preferred yeasts that enhanced their encapsulation abilities, and *S*. *cerevisiae* was neither the most preferred nor the most beneficial. Therefore, the optimal choice for protection against parasitoid wasps likely depends on whether enhancing encapsulation ability, medicating with ethanol, or exploiting another repulsive substance is most effective against a particular wasp species.

During the course of antagonistic coevolution, parasites may evolve counter-defenses that render host immune responses ineffective. However, evolutionary trajectories are expected to differ between specialist and generalist interactions because populations of specialist parasites experience more constant selection pressures to infect particular hosts over evolutionary time [5]. This is reflected in the different strategies used by the specialist wasp *L*. *boulardi* and the generalist wasp *L*. *heterotoma* to incapacitate the immune system of *D*. *melanogaster*: the specialist alters and avoids host hemocytes whereas the generalist directly attacks host hemocytes [22, 24, 25]. We predicted that the specialist wasp would be better able to withstand ethanol. Our results broadly supported this prediction, as ethanol conferred more effective protection against the generalist than the specialist. However, contrary to recent studies [31, 32], we did not find that fly larvae seek out ethanol for self-medication after being attacked by wasps, or that adult flies increase their oviposition preference for ethanol after being exposed to wasps. Instead, our results suggest that female *D*. *melanogaster* have an innate oviposition preference for ethanol, which provides their offspring with passive protection against parasitoid wasps as a side benefit. However, in natural interactions between fruits, yeasts, flies, and parasitoid wasps, substances besides ethanol can attract or repel ovipositing insects and mediate resistance against wasps. Future studies that elucidate the connections between oviposition preference and offspring performance in fly populations that interact with different fruit, yeast, and wasp species will allow us to better understand the evolution of behavioral defenses against parasitoid wasps.

## Supporting information

S1 Appendix Results from additional larval food preference and adult oviposition preference experiments. (DOCX)

S1 Dataset Raw data corresponding to Fig 1. Eclosion outcomes of unparasitized fly larvae fed 0% or 6% ethanol food in the 48 hours after reaching second instar.
(XLSX)

S2 Dataset Raw data corresponding to Fig 2. Attack rates, attack intensities, and eclosion outcomes when fly larvae were fed 0% or 6% ethanol food in the 24 hours before or after exposure to wasps.
(XLSX)

S3 Dataset Raw data corresponding to Fig 3. Attack rates, attack intensities, and eclosion outcomes when fly larvae were placed in 0% or 6% ethanol food, then immediately exposed to wasps.
(XLSX)

S4 Dataset Raw data corresponding to Fig 4. Number of larvae (exposed or unexposed to wasps) that had migrated to the 0% and 6% ethanol sides of bisected Petri dishes by the end of 24-hour food choice assays, along with attack rates for samples of wasp-exposed larvae.
(XLSX)

S5 Dataset Raw data corresponding to Fig 5. Eggs laid on 0% ethanol food (eggs0) and 6% ethanol food (eggs6) by female flies (exposed or unexposed to wasps) during 24-hour oviposition choice assays.
(XLSX)
