# Supplementary material for: Ethanol confers differential protection against generalist and specialist parasitoids of Drosophila melanogaster
Source: PLoS One. 2017 Jul 12;12(7):e0180182. doi: 10.1371/journal.pone.0180182 (PMC5507509; doi:10.1371/journal.pone.0180182)
Supplement: S1 Appendix — (DOCX) [file pone.0180182.s001.docx]

**S1 Appendix. Results from additional larval food preference and adult oviposition preference experiments.**

**Ethanol Confers Differential Protection Against Generalist and Specialist Parasitoids of *Drosophila melanogaster***

Zachary R. Lynch, Todd A. Schlenke, Levi T. Morran, and Jacobus C. de Roode

**Larval ethanol food preference**

The results below are from our first larval food preference experiment, in which we did not measure ambient light, temperature, or humidity conditions. We later decided that it was necessary to run a 15 h light: 9 h dark cycle and measure temperature and humidity conditions in the lab, so we repeated this experiment. Otherwise, we used the same protocol described in the Materials and Methods in the main text.

*D. melanogaster* larvae that were exposed to wasps did not show increased migration from the 0% ethanol side to the 6% ethanol side of bisected Petri dishes during 24 h food choice experiments (Fig S1a; *F_2,6_* = 2.2, *P* = 0.19). Exposure to wasps also had no effect on the propensity of fly larvae to stay in 6% ethanol food when they started there (Fig S1b; *F_2,6_* = 0.42, *P* = 0.68). The overall tendency, regardless of wasp exposure treatment and starting side, was to stay on the starting side (*t_17_* = 12.0, *P* < 0.0001). Lack of wasp exposure effects cannot be explained by low attack rates: 82.8 ± 0.08% and 93.3 ± 0.03% of larvae exposed to *L. boulardi* and *L. heterotoma* (respectively) contained wasp eggs.

**Fig S1.** **Larval ethanol food preference.** Proportion of late second to early third-instar *D. melanogaster* larvae in three wasp exposure treatments (unexposed controls, exposed to *L. boulardi* (Lb17), and exposed to *L. heterotoma* (Lh14)) that were on the 6% ethanol side at the end of a 24 h choice experiment after starting on the 0% ethanol side **(a)** or the 6% ethanol side **(b)** of bisected Petri dishes (3 replicates per wasp exposure and starting side combination, ~70 larvae per replicate, error bars: ± 1 SEM).

**Adult ethanol oviposition preference**

The results below are from our first set of oviposition preference experiments, in which we maintained constant 24 h overhead light and did not measure temperature or humidity. We later decided that it was necessary to run a 15 h light: 9 h dark cycle and measure ambient temperature and humidity conditions in the lab, so we repeated these experiments. Otherwise, we used the same population cage protocol described in the Materials and Methods in the main text.

We conducted ethanol oviposition preference experiments in large population cages using two methods of preparing the 6% ethanol food. In the trials with well-mixed food, we observed no significant preference for either 0% or 6% ethanol oviposition sites (Fig S2a; *t_23_* = 1.2, *P* = 0.23) and no effect of wasp treatment (*F_2,21_* = 1.8, *P* = 0.19). In the trials with ethanol-on-top food, flies showed a significant oviposition preference for 6% ethanol (Fig S2b; *t_23_* = 7.5, *P* < 0.0001) and there was no effect of wasp treatment (*F_2,21_* = 0.15, *P* = 0.86). For both experiments, data were pooled across the two choice periods (0–24 and 24–48 h) because there was no effect of choice period on oviposition preference (*F_1,20_* < 0.23, *P* > 0.64) and no significant interaction between choice period and wasp treatment (*F_2,18_* < 0.64, *P* > 0.54).

**Fig S2. Adult ethanol oviposition preference.** Proportion of eggs laid on 6% ethanol food when female flies were allowed to choose between 0% and 6% ethanol food in the absence of wasps (control) or in the presence of female *L. boulardi* (Lb17) or *L. heterotoma* (Lh14) using population cages. Two methods of preparing the 6% ethanol dishes were compared: thoroughly mixing ethanol into the food **(a)** and pipetting 1 mL 95% ethanol over the food after 15 mL red 0% ethanol solution had been added **(b)**. N = 8 per treatment, error bars: ± 1 SEM.
